# The cerebellum and anxiety

**DOI:** 10.3389/fncel.2023.1130505

**Published:** 2023-02-22

**Authors:** Pei Wern Chin, George J. Augustine

**Affiliations:** Program in Neuroscience & Mental Health, Lee Kong Chian School of Medicine, Nanyang Technological University, Singapore, Singapore

**Keywords:** cerebellum, anxiety, Purkinje cells, molecular layer interneurons, neuromodulation

## Abstract

Although the cerebellum is traditionally known for its role in motor functions, recent evidence points toward the additional involvement of the cerebellum in an array of non-motor functions. One such non-motor function is anxiety behavior: a series of recent studies now implicate the cerebellum in anxiety. Here, we review evidence regarding the possible role of the cerebellum in anxiety—ranging from clinical studies to experimental manipulation of neural activity—that collectively points toward a role for the cerebellum, and possibly a specific topographical locus within the cerebellum, as one of the orchestrators of anxiety responses.

## Introduction

The traditional role of the cerebellum is to coordinate and refine motor activity (Thach et al., 1992; Morton and Bastian, 2004). This view stems from extensive evidence that the most discernible and predominant behavior consequences of cerebellar dysfunction are motor-related pathologies, such as motor imbalance, ataxia, and dystonia (Thach et al., 1992; Ito, 2002; Koeppen, 2018). Ample experimental evidence also implicates the cerebellum in motor learning (Nilaweera et al., 2006; Chen et al., 2022). Nevertheless, despite its prevalent role in motor behavior, the cerebellum is also known to be anatomically connected to brain regions involved in behaviors beyond motor action, opening the possibility that the cerebellum contributes to non-motor functions (Strick et al., 2009; Stoodley and Schmahmann, 2010; Fujita et al., 2020; Novello et al., 2022). Indeed, recent data indicate that the cerebellum is involved in a variety of higher-order functions such as perception, attention, emotion, and cognition (Richter et al., 2005; Schmahmann and Caplan, 2006; Schmahmann et al., 2007; Bastian, 2011; D’Angelo and Casali, 2012; Wang et al., 2014). Clinical studies have also pointed toward cerebellar involvement in numerous psychiatric disorders not traditionally thought to involve the cerebellum, such as attention deficit hyperactivity disorder, autism spectrum disorders, schizophrenia, bipolar disorder, depression, and anxiety disorders (Liao et al., 2010; Phillips et al., 2015; Talati et al., 2015; Ke et al., 2016; Depping et al., 2018; Baek et al., 2022). Evidence from both clinical and animal studies now indicates that the cerebellum is topographically organized to modulate a multitude of behaviors, ranging from primary sensorimotor functions to integrated higher-order functions, in different anatomical and functional compartments (Strick et al., 2009; Stoodley and Schmahmann, 2010; Reeber et al., 2013; Klein et al., 2016; Fujita et al., 2020; Novello et al., 2022). These studies mark a pivotal step in advancing our understanding of how the cerebellum could integrate and carry out such diverse roles.

This review will focus on the role of the cerebellum in anxiety. We provide a brief overview of anxiety and its disorders and highlight both clinical and experimental studies that support a cerebellar contribution to anxiety behavior. Recent progress in identifying a cerebellar topographical locus implicated in mediating anxiety behavior will be presented along with the current status of mapping anxiety behavior onto cerebellar compartments. The cerebellar circuits known to influence anxiety behavior will be described, along with the possible involvement of additional circuitry, in the cerebellum and beyond. Finally, we will discuss the potential contribution of neuromodulatory systems in cerebellum-related anxiety behavior.

## Cerebellar involvement in anxiety

### What is anxiety?

Before addressing the role of the cerebellum in anxiety, it is useful to define what we mean by anxiety. Anxiety is a negative valence emotion elicited by worried thoughts and tension, notably when the source of threat is uncertain or not imminent. Anxiety elicits defensive behavioral responses—such as enhanced vigilance—to anticipate perceived and potential threats that are uncertain or distant (Calhoon and Tye, 2015). Excessive and prolonged anxiety is often paired with habitually overestimating the level of danger or the perpetual need to avoid threatening situations. These dysfunctional behaviors are often used as coping mechanisms by patients with various anxiety disorders, such as Generalized Anxiety Disorder (GAD), Social Anxiety Disorder (SAD), and Panic Disorder (American Psychiatric Association, 2022). The wide range of anxiety disorders often includes common features, such as excessive fear and anxiety, often toward a specific context or object.

When discussing anxiety and fear-related studies, it is imperative to employ clear definitions and terminologies. Even though the emotional states of fear and anxiety overlap, anxiety differs notably from fear: anxiety is an emotional response generated toward uncertain future threats, whereas fear is an emotional response generated in response to real and imminent threats (Tovote et al., 2015). LeDoux and Pine (2016) have proposed that the mental states of fear and anxiety should be discussed separately from their defensive behavior and physiological responses. Thus, here we will refer to the defensive behavior generated by feelings of anxiety as “anxiety behavior”. LeDoux and Pine (2016) further suggest that the conscious mental state of anxiety may not be mediated by the same brain circuitry that is used for the behavioral and physiological responses to anxiety. However, it is evident that such circuitry, even if separate, needs to be coupled to generate behavioral and physiological responses. Additionally, because many of the studies to be discussed in our review have been performed in animal models, where it is impossible to determine whether animals consciously feel emotions, we will use the term “anxiety circuit” to refer to the interconnected brain circuits collectively responsible for anxious feelings, anxiety behavior, and anxiety physiological responses.

Despite anxiety disorders being the most prevalent type of psychiatric disorder—with a lifetime prevalence of up to 33.7% (Bandelow and Michaelis, 2015)—current therapeutic approaches to anxiety disorders remain inadequate. This is due, in part, to a limited understanding of the neuronal circuits underlying anxiety and its behavior. Previous research has established roles for several brain regions—notably the amygdala, hippocampus, and prefrontal cortex—in processing anxiety information [as reviewed by Calhoon and Tye (2015) and Tovote et al. (2015)]. However, as our review will make clear, it is likely that other brain regions, most notably the cerebellum, are also part of the neural circuitry for anxiety and may be novel potential targets for therapeutic action.

### Clinical evidence of cerebellar perturbations in mood and anxiety disorders

#### Mood

Clinical brain stimulation studies have provided indications of a role for the cerebellum in regulating emotions as well as anxiety. A single session of repetitive, high-frequency transcranial magnetic stimulation (rTMS) of the medial cerebellum elevated positive mood in healthy volunteers (Schutter et al., 2003), whereas in a follow-up study, slow-frequency rTMS of the cerebellar vermis in healthy volunteers amplified negative mood (Schutter and van Honk, 2009). Although Schutter et al. (2003) acknowledged a lack of objectivity in the quantification of mood changes in their pilot study and provided a number of alternative interpretations for their observations, they nonetheless concluded that these studies collectively indicated the possible involvement of the cerebellum in regulating emotions. Additionally, chronic cerebellar stimulation reportedly led to a reduction in “tension-anxiety” in 63% of neurologically-impaired patients and mood improvement in 38% of these patients, although 8% of patients reported worsened “tension-anxiety” after treatment (Riklan et al., 1977). Thus, the cerebellum may be a valuable target for mood and anxiety-related therapies, in particular for mood disorders (reviewed in Lupo et al., 2019) as well as fear and anxiety-related disorders (reviewed in Moreno-Rius, 2018). However, our lack of understanding of how the cerebellum may affect emotions, particularly anxiety, is a major obstacle to improving therapeutic options.

#### Anxiety disorders

Changes in cerebellar activity and connectivity have been associated with anxiety disorders in several clinical studies (Engel et al., 2009; Moreno-Rius, 2018). Most of this work is related to SAD. SAD patients exhibit increased connectivity between the cerebellum and the amygdala (Liao et al., 2010), as well as the posterior cingulate (Doruyter et al., 2016). Patients suffering from SAD have higher activity in both their cerebellum and amygdala in response to stimuli that induce social anxiety (Evans et al., 2008). Further, the volume of the left cerebellum of SAD patients is increased in comparison to healthy controls (Talati et al., 2013). A more compelling implication of the cerebellum in SAD has come from the work of Cassimjee et al. (2010), who detected decreases in left cerebellar hemisphere volume upon treatment with a selective-serotonin reuptake inhibitor (SSRI) and consequent reduction in SAD symptoms. This reversion of cerebellar volume in SAD patients correlated with the reduction in SAD symptoms, suggesting possible cerebellar involvement in the manifestation of social anxiety symptoms. Conversely, Talati et al. (2015) found an increase in the volume of the right cerebellar hemisphere volume upon SSRI treatment and associated improvement in SAD symptoms. The discrepancy in the effects of anxiolytic drug treatment on cerebellar volume may arise from different parts of the cerebellum being measured in these two studies: SAD patients have higher resting perfusion of their right cerebellum and decreased resting perfusion in their left cerebellum (Furmark et al., 2002; Warwick et al., 2008). These opposing alterations in the left and right cerebellar hemispheres of SAD patients can potentially explain the discrepancy between the findings of Cassimjee et al. (2010) and Talati et al. (2015). Further, different SSRIs were utilized in these studies: the former employed escitalopram, while the latter used paroxetine. It is possible that the varying mechanisms of action and side effects of these two drugs could have yielded contrasting observations. Nevertheless, it is evident that these studies collectively emphasize that parts of the cerebellum may be differentially engaged during anxiety, a topic discussed at length below.

The cerebellum has also been implicated in other types of anxiety disorders. Connectivity between the cerebellum and the basolateral and centromedial amygdala reportedly is increased in GAD patients, compared to healthy controls (Etkin et al., 2009; Roy et al., 2013). In addition, PD patients exhibit higher levels of cerebellar glucose utilization (Sakai et al., 2005), an effect that is reversed by treatments that reduce their PD symptoms (Prasko et al., 2004). Cerebellar changes are also associated with other mental disorders that are closely related to anxiety disorders, such as Post Traumatic Stress Disorder (Bonne et al., 2003; Ke et al., 2016) and Obsessive-Compulsive Disorder (Xu et al., 2019; Zhang H. et al., 2019; Kashyap et al., 2021). Although these conditions are considered to differ from anxiety disorders in the Diagnostic and Statistical Manual of Mental Disorders, Fifth Edition (DSM-5), their pathophysiology nonetheless can include an anxiety component (American Psychiatric Association, 2022). Taken together, it is likely that the cerebellum plays a role in the manifestation or progression of an array of clinical anxiety disorders.

Although there is progress in clinical efforts to identify the possible involvement of the cerebellum in anxiety disorders, these must be viewed as merely a promising start. Most clinical studies performed to date have relatively small sample groups, a recurring issue, especially in the field of clinical neuroimaging (Szucs and Ioannidis, 2020). Meta analyses and longitudinal studies on the relationship between cerebellar changes and anxiety disorders are sorely lacking. These shortcomings can be attributed to a lack of attention to the cerebellum, specifically in regional cerebral blood flow studies where the cerebellum is often used as a reference region due to assumptions of its irrelevance to psychiatric disorders (Bonne et al., 2003). Moreover, further effort is required to standardize the clinical measures of anxiety and its symptoms, to allow quantifiable research output and better interpretability of data (Baker et al., 2019). These systematic changes in clinical studies, though daunting, will be necessary to advance our understanding of the role of the human cerebellum in anxiety disorders.

Additionally, despite the ability of such clinical studies to implicate the cerebellum in anxiety, they cannot determine whether cerebellar abnormalities produce the pathophysiology of anxiety, or whether these abnormalities result from anxiety. To understand the possible causal relationships between cerebellar changes and anxiety, it is essential to turn to experimentation in animal models of anxiety. When considering such work, it is important to acknowledge that studies of emotions based on animal models must rely on behavioral and physiological proxies of their mental state, which necessarily limits the interpretations that can be derived from such studies. Despite such limitations, animal experiments have greatly expanded our knowledge of the role of the cerebellum in anxiety.

### Early experimental evidence for a cerebellar role in anxiety behavior

#### Rodent cerebellar lesion studies

**Figure 1:**
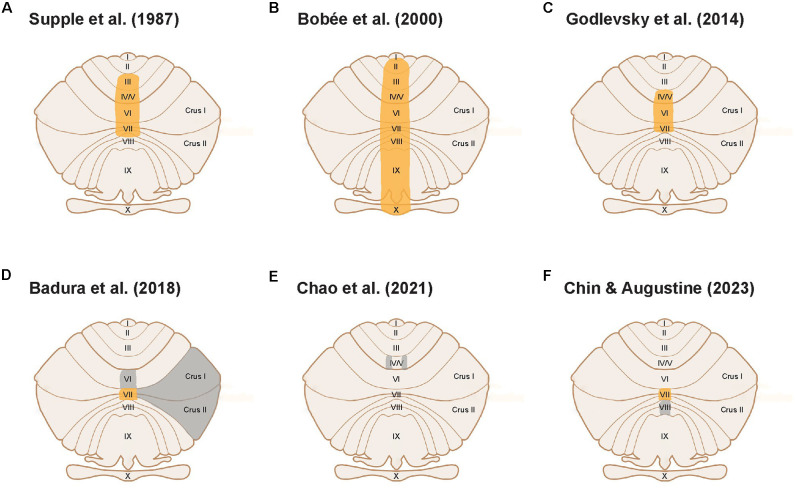
Cerebellar cortical areas experimentally tested for a functional role in anxiety behavior. Unfolded view of the cerebellar cortex illustrates cerebellar lobules. Orange areas indicate cerebellar lobules implicated in anxiety behavior; gray areas indicate cerebellar areas that were not implicated in anxiety behavior. **(A–C)** Large-scale lesion or electrical stimulation studies establishing a cerebellar role in anxiety. **(D)** Lobule VII modulates anxiety behavior, whereas lobules VI, Crus I, and II do not. **(E)** Areas within lobules IV/V do not affect anxiety behavior. **(F)** Lobule VII modulates anxiety while lobule VIII does not.

The first suggestion of a possible role for the cerebellum in emotional behavior came from cerebellar lesion experiments (Chambers and Sprague, 1955). The first experimental evidence for cerebellar involvement in anxiety behavior came later: Supple et al. (1987) reported that lesions of the cerebellar vermis of rats (Figure 1A) exhibited greater anxiety behavior, indicated by an aversion to the central area of an open field (thigmotaxis). Numerous subsequent cerebellar lesion studies have supported this finding. For example, Bobée et al. (2000) observed increases in the number of entries and time spent in open arms of the elevated-plus maze (EPM) in young vermis-lesioned rats (Figure 1B), also indicating an anxiolytic effect of vermal lesions. Collectively, such studies established a plausible role for the cerebellum in anxiety behavior.

#### Cerebellar disease model: the lurcher mouse

Another indication of the possible involvement of the cerebellum in anxiety behavior comes from work done on the Lurcher mutant mouse, a cerebellar disease model. Lurcher mice have a mutation in the δ2 glutamatergic subunit, which is a receptor for molecules (cerebellins) involved in synapse formation (Uemura et al., 2010). Mutation of this receptor leads to the complete loss of Purkinje cells in the cerebellar cortex. This, in turn, yields retrograde degeneration of Purkinje cell afferents, namely cerebellar granule cells and molecular layer interneurons, as well as projection neurons from the inferior olive (Vogel et al., 2007). In addition to the motor problems indicated by their name, Lurcher mice display “behavioral disinhibition”, evident as them spending more time in the open arms of the EPM compared to non-mutant mice (Monnier and Lalonde, 1995; Hilber et al., 2004; Lorivel et al., 2010). This apparent reduction in anxiety levels is not caused by motor deficits, because Lurcher mice spend more time in the open arms of the maze and enter the open arms as frequently as, or perhaps more frequently, than control mice. Furthermore, although it is also reported that Lurcher mice have higher stress levels due to the overactivation of their hypothalamic-pituitary-adrenal (HPA) axis, experimentally reducing blood corticosterone levels does not abolish the low anxiety state. This indicates that the anxiolytic behavior displayed by Lurcher mice may arise from disruptions in the neural circuitry involved in regulating anxiety behavior (Lorivel et al., 2010).

#### Spinocerebellar ataxia rodent models

Studies on spinocerebellar ataxia (SCA) rodent models also support a cerebellar role in anxiety behavior. SCA models are generally characterized by abnormal expansion of CAG repeats in genes that lead to an ataxic pathology due to neurodegeneration in the cerebellum, often in Purkinje cells (Boy et al., 2009, 2010; Kelp et al., 2013; Asher et al., 2021; Bohne et al., 2022). Anxiety behavior is one of the most studied behaviors amongst the various non-sensorimotor impairments also observed in SCA models, beyond their characteristic ataxic phenotype. Many SCA models exhibit anxiolytic behavior (Boy et al., 2009), although anxiogenic behavior is also often observed (Kelp et al., 2013). Interestingly, in certain SCA models, both abnormalities in anxiety behavior could be observed by varying the expression pattern of the mutant gene, or by simply modifying the aversiveness of the external environment. For instance, Asher et al. (2021) demonstrated that SCA 1 knock-in mice display anxiogenic traits, whereas Purkinje cell-specific SCA 1 knock-in mice exhibit anxiolytic behavior. Additionally, Bohne et al. (2022) showed that in their SCA 6 mouse model, reduced anxiety was observed in low-threat conditions, but these mice displayed enhanced anxious defensive behaviors in contexts with higher aversiveness. This suggests a possible miscalculation of threat probability due to cerebellar pathology. Collectively, the SCA rodent models provide further support for a possible cerebellar involvement in regulating anxiety behavior.

## Topographical locus of the cerebellar circuitry modulating anxiety

Both clinical and experimental evidence strongly suggests a functional role for the cerebellum in anxiety and anxiety behaviors. In addition, the cerebellum is also implicated in an array of motor and non-motor functions, which leads to the question of whether these functions are carried out by the same or different regions of the cerebellum. If the cerebellum does spatially compartmentalize these functions, it would be valuable to pinpoint which cerebellar regions and local circuits are responsible for mediating anxiety and its behavior.

### A functional locus in the cerebellar cortex for anxiety behavior

As discussed above, early lesion studies focused on the cerebellar vermis as a locus for anxiety behavior in rodents (Supple et al., 1987; Bobée et al., 2000). A handful of clinical studies have also correlated anxiety disorders in humans with abnormalities in vermis activity and vermis-amygdala connectivity (Liao et al., 2010; Ke et al., 2016). Godlevsky et al. (2014) further localized the posterior vermis as the region involved in modulating anxiety behavior by showing that electrical stimulation of cerebellar lobules V-VII in kindled rats had an anxiolytic effect (Figure 1C). This is consistent with the consensus that the posterior vermis is involved in emotion processing (Klein et al., 2016), in this case, anxiety behavior.

The studies described thus far targeted large areas of the cerebellum by employing lesions, genetic models, or electrical stimulation. A recent study has initiated a more precise definition of the cerebellar anatomical compartments involved in anxiety behavior (Badura et al., 2018). This study used targeted expression of an inhibitory chemogenetic receptor to silence molecular layer interneurons (MLIs) in different compartments within the posterior vermis of adult mice. Badura et al. (2018) found that acute inhibition of MLIs in lobule VII increased time spent in the open arms of the EPM, compared to control mice. Interestingly, developmental inhibition of MLIs in lobule VII instead decreased time spent in the open arms of the EPM when compared to relevant controls. Further research will be required to clarify the opposing effects of developmental and adult manipulation of lobule VII MLIs on EPM behavior observed in this study. Nevertheless, this work points towards a role for lobule VII in regulating EPM-related behavior. In contrast, inhibition of MLIs in lobule VI, Crus I, or Crus II in adult mice had no significant effect on exploratory time in the open arms of EPM when compared to control mice. Although the authors interpreted the behavioral effects of lobule VII MLI manipulation as impairment of novelty-seeking and exploratory behavior, an alternative explanation is that manipulation of lobule VII MLI activity affects anxiety behavior. The EPM is conventionally used as a test of anxiety behavior, by employing an animal’s innate and conflicting drives to explore novel spaces while avoiding possibly threatening spaces (Calhoon and Tye, 2015), The lack of a significant effect of MLI inhibition in lobule VI, Crus I and Crus II indicates that lobule VII has a unique role in regulating anxiety behavior (Figure 1D). This conclusion is further supported by a recent study showing that manipulation of specific areas within lobules IV/V did not affect anxiety behavior (Figure 1E; Chao et al., 2021).

**Figure 2:**
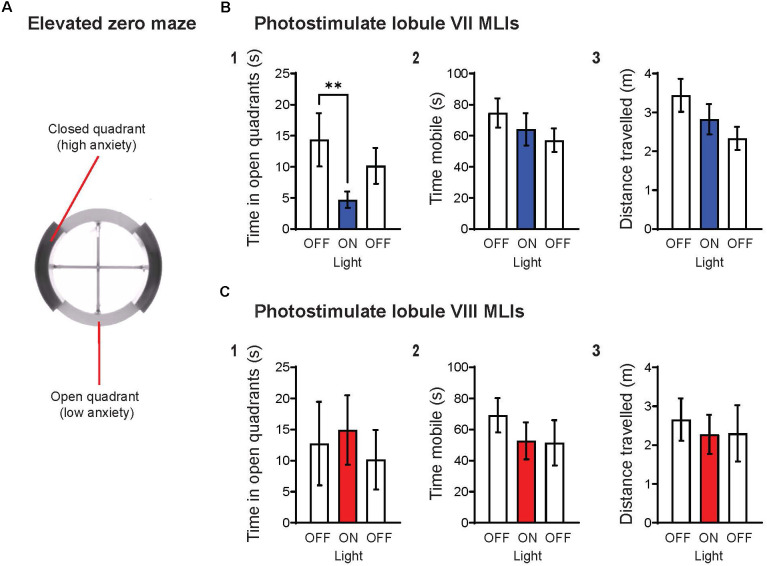
Optogenetic stimulation of MLIs in lobule VII, but not lobule VIII, increases anxiety levels. **(A)** Schematic representation of the elevated zero maze (EZM), consisting of two open quadrants (45 lux) and two closed quadrants (15 lux) enclosed by 14 cm high walls. The EZM has an outer diameter of 55 cm, a 6 cm-wide platform, and is elevated 60 cm off the floor. Animals were tested on the EZM for a total of 6 min, with three 2-min epochs OFF-ON-OFF, where light (470 nm, 11 mW/mm^2^, 10 Hz, 50% duty cycle) was delivered only during the “ON” epoch. Timed light delivery and behavioral tracking were performed using the ANY-maze software. Upon completion of experiments, histological confirmation of genotype and optical fiber placement were performed for all animals. Animals with indeterminate or incorrect placement of optical fiber were excluded from the analysis. **(B)** Photostimulation of lobule VII MLIs (1) significantly decreased time spent in open quadrants (*p* = 0.008**), yet had no effect on (2) mobility or (3) distance traveled. **(C)** Photostimulation of lobule VIII MLIs did not have any significant effects on the indicated behaviors. Wilcoxon signed rank test; lobule VII: *n* = 8; lobule VIII, *n* = 5. Data are mean ± SEM.

Our recent experiments provide complementary evidence for a specific role for lobule VII in the anxiety circuit, by demonstrating that acute activation of MLIs in this lobule produces an anxiogenic effect. In brief, we regionally photostimulated MLIs in adult transgenic mice expressing the light-activated cation channel, channelrhodopsin-2 (ChR2), exclusively in MLIs (Kim et al., 2014). Optogenetic stimulation of MLIs in lobule VII significantly decreased the amount of time spent in the open quadrants of the elevated-zero maze (EZM; Figures 2A,B1), which is an improved variant of the EPM (Braun et al., 2011; Tucker and McCabe, 2017). This effect was not accompanied by changes in the time that the mice were mobile (Figure 2B2) or the distance that the mice traveled (Figure 2B3), indicating that photostimulation of lobule VII MLIs increased anxiety levels rather than altering motor function. Further, photostimulation of MLIs in lobule VIII did not evoke anxiety behavior (Figure 2C), nor did illumination of either lobule in mice not expressing ChR2 in MLIs (not shown). In summary, these optogenetic analyses further establish cerebellar lobule VII as a specific topographical locus for anxiety behavior (Figure 1F). The possible roles of most other cerebellar lobules in anxiety have yet to be tested.

It is widely accepted that the fear and anxiety circuits overlap extensively (Calhoon and Tye, 2015; Tovote et al., 2015), although there is also a notable divergence between the two. The lack of an effect on anxiety behavior upon activation of MLIs in lobule VIII (Figure 2C), as well as inhibition of MLIs in lobule VI (Badura et al., 2018), indicates a topographical divergence of the fear and anxiety circuits within the cerebellar cortex: lobules VI and VIII are known for their roles in fear responses (Sacchetti et al., 2002, 2007; Zhu et al., 2007; Koutsikou et al., 2014). Thus, it appears that lobule VII is involved in anxiety behavior, while lobules VI and VIII are involved in fear behavior. The discovery of spatial divergence within the cerebellar cortex between anxiety and fear-related behaviors may prove to be an exciting breakthrough for both the cerebellum and fear/anxiety fields.

#### Topographical map hypotheses and the anatomical link of the cerebellar cortex to the anxiety circuit

The identification of lobule VII as a functional locus for anxiety behavior aligns with current hypotheses about the topographical organization of the cerebellum. One hypothesis postulates that the posterior cerebellum modulates non-motor behavior (Stoodley and Schmahmann, 2010, 2018), while another suggests that the vermis regulates emotion (Timmann et al., 2010; Klein et al., 2016). The module hypothesis posits that the cerebellar functions are spatially organized in “modules”, areas organized according to their connectivity and outputs (Apps et al., 2018). These modules could extend across multiple lobules (Fujita et al., 2020). Notably, each of the vermal lobules I-IX comprises a multitude of modules, and each lobule could be involved in multiple functions. Lobule VII, located in the posterior vermis, is anatomically connected to functional circuits that are involved in threat, behavioral flexibility, arousal, sensory-motor gating, and cardiorespiratory functions (Fujita et al., 2020), which are vital in processing and producing defensive responses, such as anxiety. It is possible that cerebellar areas in addition to lobule VII could be part of an anxiety module, due to the abundant anatomical connections between the cerebellum and other anxiety-related brain regions. However, we do know that perturbation of parts of lobules IV/V, as well as lobules VI, VIII, Crus I, and II, does not evoke anxiety behavior (Figure 1; Badura et al., 2018; Chao et al., 2021).

The cerebellar vermis is known as the “limbic cerebellum” because of its numerous anatomical connections to the rest of the limbic system (Blatt et al., 2013; Reeber et al., 2013). These include brain regions that are known to mediate anxiety, such as the periaqueductal gray (PAG; Snider and Maiti, 1976) and dorsal raphe nuclei (DRN; Pierce et al., 1977). These connections provide a means for the cerebellum, in particular lobule VII, to communicate with the rest of the brain anxiety circuitry. Additionally, the well-established bidirectional connection between the cerebellum and hypothalamus (Dietrichs, 1984; Dietrichs and Haines, 1986; Supple, 1993; Kondo et al., 1998; Cavdar et al., 2018) could possibly underlie a cerebellar autonomic modulation during periods of fear or anxiety. Neurophysiological and neuroimaging studies support a cerebellar role in regulating visceral activities, such as gastrointestinal motor control and cardiovascular modulation (Zhu et al., 2006; Schutter, 2012; Ito and Nisimaru, 2014). This pathway provides a direct anatomical link between the cerebellum and the HPA axis that is critical for the physiological responses of fear and anxiety, such as heart rate, breathing rate, and stress hormone regulation. Because lobule VII includes a module governing cardiorespiratory functions (Fujita et al., 2020), it is possible that this anatomical connection could also involve lobule VII in anxiety-related physiological responses.

### The deep cerebellar nuclei and anxiety behavior

Downstream from the cerebellar cortex, the deep cerebellar nuclei (DCN) are the sole output stations of the cerebellum. The DCN consists of three sets of nuclei: lateral, interposed, and medial. A handful of studies have examined the involvement of the DCN in anxiety. Bauer et al. (2011) demonstrated that bilateral electrolytic lesions of the lateral DCN did not influence anxiety behavior in rats when tested on the EPM. Zhang Q. et al. (2019) reported that activation of the lateral and interposed DCN in mice subjected to photothrombotic strokes also did not affect anxiety behavior. On the other hand, a more recent study performed by Frontera et al. (2020) demonstrated that the medial DCN regulates anxiety behavior. Activation of medial DCN neurons projecting to the parafascicular (PF) thalamus, but not to the ventrolateral PAG (vlPAG), had an anxiogenic effect, reflected by decreased number of entries into the open arms of the EPM as well as decreased number of entries into the light zone in the light-dark box. Frontera et al. (2020) also revealed a divergence of the fear and anxiety circuits within the medial DCN: medial DCN-vlPAG projections are involved in fear behavior, whereas medial DCN-PF thalamic projections modulate anxiety behavior. This finding complements the topographical divergence of anxiety and fear seen within the cerebellar cortex, suggesting that the topographical organization of cerebellar functions extends from the cerebellar cortex into the DCN.

## Cerebellar neurons and circuits in anxiety

Now that anxiety loci within the cerebellum have been identified, we are poised to explore the local circuits within these loci that are involved in mediating anxiety behavior. The goal will be to investigate how cerebellar circuitry receives, processes, and relays information that ultimately mediates anxiety behavioral and physiological responses. From a handful of initial studies, we already know that the MLIs, Purkinje cells (PCs), and granule cells (GCs) of the cerebellar cortex are involved in the cerebellar modulation of anxiety behavior, as are a subpopulation of glutamatergic neurons in the medial DCN. This section will summarize the information currently available.

### Cerebellar cortex

#### Neurons

Alteration of MLIs activity in lobule VII bidirectionally controls anxiety behavior. As discussed above, chemogenetic inhibition of these MLIs has an anxiolytic effect (Badura et al., 2018), while their optogenetic activation has an anxiogenic effect (Figure 2B). Additionally, cerebellar cortical GCs also play a role in cerebellar-related anxiety behavior. Rudolph et al. (2020) demonstrated that genetic deletion of the δ GABA_A_ receptor subunit in GCs, to eliminate GABAergic tonic inhibition and make GCs hyperexcitable, increases anxiety levels.

PCs are the sole output neurons of the cerebellar cortex (Ito, 2006). Because MLIs and GCs provide direct input to PCs, PCs must relay anxiety information from the cerebellar cortex to the DCN. Indeed, mice display anxiogenic behavior when Shank2, an excitatory postsynaptic scaffolding protein, is knocked out of PCs, an effect not observed in global Shank2 knockout mice which instead exhibit autistic-like behaviors (Ha et al., 2016). Shank2 clusters were found to colocalize extensively with the excitatory synaptic vesicular glutamate transporter 1 (VGluT1), which is expressed selectively in parallel fibers as opposed to climbing fibers (Hioki et al., 2003), indicating postsynaptic localization of Shank2 at the PF-PC synapses This is associated with a decrease in the frequency of spontaneous excitatory synaptic currents in PCs, suggesting a net reduction of GC excitatory input into PCs that affects anxiety behavior. Thus, both inhibitory and excitatory inputs into PCs, as well as their effect on PC output, seem to play a significant role in anxiety behavior processing within the cerebellar cortex.

#### Circuits

The effects of these cell-level effects on cerebellar cortex circuity are complex. The results above suggest that increasing PC activity—*via* MLI inhibition and consequent PC disinhibition—will decrease anxiety levels. Conversely, decreasing PC activity—*via* MLI activation or decreasing excitatory input into PCs—should increase anxiety levels. However, there are other experimental observations that do not fit into this simple scheme. For example, GC hyperexcitability should increase PC activity, yet it increases anxiety instead of the expected decrease (Rudolph et al., 2020). The complex nature of neuronal connectivity and interactions upstream of PCs presumably account for such contradictions. Despite having an overall inhibitory effect on PCs, MLIs also inhibit each other *via* chemical synapses and excite each other through electrical synapses (Kim et al., 2014; Rieubland et al., 2014). Moreover, while having an overall excitatory effect on PCs, GCs also excite interneurons—MLIs as well as Golgi cells—which in turn would inhibit GCs and decrease PC output (Dieudonne, 1998; Dugué et al., 2005; Kim and Augustine, 2021). Therefore, manipulation of GC and MLI activity will not simply translate to changes in PC activity.

In addition, PCs also directly receive or are indirectly modulated by an array of afferents originating from outside the cerebellar cortex, including mossy fibers, climbing fibers, and various neuromodulator fibers (Bloedel, 1973; Longley et al., 2021; Canton-Josh et al., 2022). Different PC firing patterns—simple spikes evoked by mossy fiber input to parallel fibers or complex spikes resulting from climbing fiber excitation of PCs—can encode different functions (Popa et al., 2019). Because mossy fibers innervate granule cells (Ito, 2006), which are known to modulate cerebellar-related anxiety behavior (as discussed above), it is likely that mossy fiber input into the cerebellum could also be involved in cerebellar anxiety. It remains unknown whether climbing fiber inputs onto PCs could be involved in relaying anxiety information into the cerebellum.

Taken together, it is likely that complex cerebellar circuit effects upstream of PCs are involved in producing the anxiety behavioral response. How these upstream effects are relayed onto the Purkinje cells, and ultimately to the DCN, and thence translated into anxiety behavior, remains unknown. Direct experimental control of PC activity, to bypass upstream neurons in the cerebellar cortical circuitry, would help to clarify the role of PCs and the entire cerebellar cortex in anxiety.

### Deep cerebellar nuclei neurons

The involvement of DCN in mediating anxiety behavior was described in the preceding section. Some progress has been made in identifying the DCN neuron types that are responsible for these effects. Activation of a subpopulation of glutamatergic medial DCN neurons that project to PF increases anxiety levels, indicating that excitatory neurons within the DCN modulate cerebellar-related anxiety behavior (Frontera et al., 2020). However, not all glutamatergic neurons in the medial DCN modulate anxiety: a subpopulation of glutamatergic medial DCN neurons that projects to vlPAG does not affect anxiety behavior. It is currently unclear whether still other neurons within the DCN, specifically GABAergic neurons, also participate in anxiety behavior. Other subpopulations of medial DCN neurons that project to other anxiety brain regions could also be involved in mediating anxiety behavior.

## Neuromodulation of the cerebellum in anxiety

Neuromodulator transmitter systems play an important role in the manifestation of anxiety and its disorders (Cassano et al., 2002; Martin et al., 2009). Despite their importance, the role of neuromodulators in the cerebellum has been largely overlooked until recently. Despite the lack of direct evidence connecting cerebellar neuromodulator action to anxiety, by analogy to other brain areas it is likely that neuromodulators are indeed important for cerebellar function in anxiety behavior. For this reason, we will briefly mention several neuromodulators that are prime targets for future analysis.

### Histamine

Various histaminergic receptors in the cerebellar vermis have been implicated in emotional memory (Gianlorenco et al., 2011, 2012; Costa et al., 2013; Fernandes et al., 2017). This series of experiments established the role of the histaminergic system in lobules IV/V on anxiety-related emotional memory consolidation *via* the actions of histaminergic receptors H1, H3 and H4. Because these experiments investigated anxiety-related emotional memory, it is also possible that the anxiety state of the animals—rather than memory—could have been affected by the modulatory actions of histamine. Therefore, future studies determining whether the cerebellar histaminergic system specifically affects innate anxiety behavior may prove to be fruitful.

### Serotonin

Serotonin, also known as 5-hydroxytryptamine or 5-HT, is well-known for its role in regulating anxiety and its behavior in both clinical and experimental studies (Moulédous et al., 2018; Abela et al., 2020; Zangrossi et al., 2020). SSRIs are also widely utilized as a pharmacological treatment for anxiety disorders (Cassano et al., 2002). In the cerebellum, direct evidence supporting a role for serotonergic modulation of the DCN in stress-induced dystonia was recently reported (Kim et al., 2021). The involvement of cerebellar 5-HT in this aversive behavior makes it possible that 5-HT could be involved in other aversive behaviors involving the cerebellum, such as anxiety or fear.

### Other neuromodulators

Additional neuromodulators, such as dopamine and norepinephrine, are known to regulate cerebellar local circuitry. The cerebellar dopaminergic system has been shown to regulate reward-related information processing (Carta et al., 2019; Cutando et al., 2022). On the other hand, a role for norepinephrine has been established in cerebellar eyeblink conditioning (Gould, 1998; Cartford et al., 2004). While these neuromodulators are also known to participate in anxiety behavior in other brain regions (Martin et al., 2009; McCall et al., 2017; Berry et al., 2019; Morel et al., 2022), it remains to be determined whether they are involved in cerebellar-related anxiety behaviors.

## Conclusions

Although our survey of the literature reveals a substantial body of evidence implicating the cerebellum in anxiety and anxiety behavior, it is equally clear that this field is in a very early stage of development and has many knowledge gaps. Here we summarize some of the areas requiring immediate further analysis.

### Cerebellar mechanisms

We are lacking strong experimental evidence coupling specific cellular, molecular, and neuromodulator systems in the cerebellum to anxiety and anxiety behavior. The chronic, large-scale manipulations of the cerebellum performed thus far are unable to provide precise evidence regarding the cerebellar neurons and circuits involved in anxiety behavior. Moreover, it is possible that a global, diffuse approach to cerebellar targeting could produce no observable effects on anxiety behavior because of counterbalancing effects, such as the removal of both excitatory and inhibitory circuits. For instance, there could be a number of explanations for the lack of change in anxiety behavior upon a lesion of the lateral DCN by Bauer et al. (2011). While the simplest deduction is that the lateral DCN is not involved in the cerebellar anxiety circuitry, it is possible that electrolytic ablation of all lateral DCN neurons—both excitatory and inhibitory—could yield no net effect on anxiety output. It is also likely that other circuitry within or outside of the cerebellum could compensate for the removal of this part of the circuit. Another example comes from the medial DCN: despite the plethora of lesion studies performed in this part of the cerebellum. The only evidence for a role of the medial DCN in anxiety behavior came recently (Frontera et al., 2020). It is possible that the reasons listed above could have confounded earlier studies, or perhaps previous studies simply did not consider the effects of medial DCN lesions on anxiety behavior.

### Cerebellar compartments

Clearly the cerebellum is a heterogeneous structure with a multitude of functions. To yield clear results, future studies of the involvement of cerebellar circuits and neuromodulators in anxiety must target specific compartments within the cerebellum. In particular, because lobule VII and the medial DCN have been identified as cerebellar loci for anxiety, more focus must be placed on studying the local circuits within these loci and determining how they interact with other anxiety-related brain regions. Further, we would like to emphasize that experimental analyses of cerebellar compartments involved in anxiety behavior to date were not intended to be exhaustive; it is possible, indeed likely, that other cerebellar regions may be involved in mediating anxiety behavior.

### Cerebellar circuits and anxiety

Finally, while we know that the activity of lobule VII MLIs bidirectionally influences anxiety behavior, we still have no idea how other neurons in this lobule work in conjunction with the MLIs during anxiety behavior, how information is transmitted from MLIs and translated into Purkinje cell output, and subsequently transmitted to the DCN. All these questions are important and must be answered. There is currently no evidence about the other brain areas that transmit anxiety-related information to lobule VII MLIs, although anatomical connections with anxiety-related brain regions such as the PAG and DRN have been identified.

In conclusion, we hope that this review will inspire further interest in the role of the cerebellum in anxiety. The questions that we have posed should kickstart the next generation of experimental work that will provide clearer insights into how the cerebellum fits into the brain anxiety circuitry. Such work, in turn, may enable development of improved therapeutic options for human anxiety disorders.

## Data availability statement

The original data included in the article are available by directing inquiries to the corresponding author.

## Ethics statement

The animal procedures employed in the experiments shown in Figure 2 were reviewed and approved by Institutional Animal Care and Use Committee, Nanyang Technological University.

## Author contributions

Both authors provided ideas, constructed the figures, and wrote the article. All authors contributed to the article and approved the submitted version.

## References

[B1] Abela A. R. Browne C. J. Sargin D. Prevot T. D. Ji X. D. Li Z.. (2020). Median raphe serotonin neurons promote anxiety-like behavior via inputs to the dorsal hippocampus. Neuropharmacology 168:107985. 10.1016/j.neuropharm.2020.107985 32035145

[B2] American Psychiatric Association (2022). Diagnostic and Statistical Manual of Mental Disorders, *Text Revision (DSM-5-TR)*. Washington, DC: American Psychiatric Association.

[B3] Apps R. Hawkes R. Aoki S. Bengtsson F. Brown A. M. Chen G.. (2018). Cerebellar modules and their role as operational cerebellar processing units. Cerebellum 17, 654–682. 10.1007/s12311-018-0952-3 29876802 PMC6132822

[B4] Asher M. Rosa J.-G. Cvetanovic M. (2021). Mood alterations in mouse models of spinocerebellar ataxia type 1. Sci. Rep. 11, 713–713. 10.1038/s41598-020-80664-9 33436887 PMC7803946

[B5] Badura A. Verpeut J. L. Metzger J. W. Pereira T. D. Pisano T. J. Deverett B.. (2018). Normal cognitive and social development require posterior cerebellar activity. eLife 7:e36401. 10.7554/eLife.36401 30226467 PMC6195348

[B6] Baek S. J. Park J. S. Kim J. Yamamoto Y. Tanaka-Yamamoto K. (2022). VTA-projecting cerebellar neurons mediate stress-dependent depression-like behaviors. eLife 11:e72981. 10.7554/eLife.72981 35156922 PMC8843095

[B7] Baker A. Simon N. Keshaviah A. Farabaugh A. Deckersbach T. Worthington J. J.. (2019). Anxiety symptoms questionnaire (ASQ): development and validation. Gen. Psychiatry 32:e100144. 10.1136/gpsych-2019-100144 31922090 PMC6936972

[B8] Bandelow B. Michaelis S. (2015). Epidemiology of anxiety disorders in the 21st century. Dialogues Clin. Neurosci. 17, 327–335. 10.31887/DCNS.2015.17.3/bbandelow 26487813 PMC4610617

[B9] Bastian A. J. (2011). Moving, sensing and learning with cerebellar damage. Curr. Opin. Neurobiol. 21, 596–601. 10.1016/j.conb.2011.06.007 21733673 PMC3177958

[B10] Bauer D. J. Kerr A. L. Swain R. A. (2011). Cerebellar dentate nuclei lesions reduce motivation in appetitive operant conditioning and open field exploration. Neurobiol. Learn. Mem. 95, 166–175. 10.1016/j.nlm.2010.12.009 21195786

[B11] Berry A. S. White R. L. Furman D. J. Naskolnakorn J. R. Shah V. D. Esposito M.. (2019). Dopaminergic mechanisms underlying normal variation in trait anxiety. J. Neurosci. 39, 2735–2744. 10.1523/JNEUROSCI.2382-18.2019 30737306 PMC6445999

[B12] Blatt G. J. Oblak A. L. Schmahmann J. D. (2013). “Cerebellar connections with limbic circuits: anatomy and functional implications,” in Handbook of the Cerebellum and Cerebellar Disorders, eds Manto M. Schmahmann J. D. Rossi F. Gruol D. L. Koibuchi N. (Dordrecht: Springer Netherlands), 479–496.

[B13] Bloedel J. R. (1973). Cerebellar afferent systems: a review. Prog. Neurobiol. 2, 3–68. 10.1016/0301-0082(73)90006-3 4598179

[B14] Bobée S. Mariette E. Tremblay-Leveau H. Caston J. (2000). Effects of early midline cerebellar lesion on cognitive and emotional functions in the rat. Behav. Brain Res. 112, 107–117. 10.1038/s41598-023-28259-y 10862941

[B15] Bohne P. Rybarski M. Mourabit D. B.-E. Krause F. Mark M. D. (2022). Cerebellar contribution to threat probability in a SCA6 mouse model. Hum. Mol. Genet. 31, 3807–3828. 10.1093/hmg/ddac135 35708512 PMC9652111

[B16] Bonne O. Gilboa A. Louzoun Y. Brandes D. Yona I. Lester H.. (2003). Resting regional cerebral perfusion in recent posttraumatic stress disorder. Biol. Psychiatry 54, 1077–1086. 10.1016/s0006-3223(03)00525-0 14625150

[B17] Boy J. Schmidt T. Schumann U. Grasshoff U. Unser S. Holzmann C.. (2010). A transgenic mouse model of spinocerebellar ataxia type 3 resembling late disease onset and gender-specific instability of CAG repeats. Neurobiol. Dis. 37, 284–293. 10.1016/j.nbd.2009.08.002 19699305

[B18] Boy J. Schmidt T. Wolburg H. Mack A. Nuber S. Böttcher M.. (2009). Reversibility of symptoms in a conditional mouse model of spinocerebellar ataxia type 3. Hum. Mol. Genet. 18, 4282–4295. 10.1093/hmg/ddp381 19666958

[B19] Braun A. A. Skelton M. R. Vorhees C. V. Williams M. T. (2011). Comparison of the elevated plus and elevated zero mazes in treated and untreated male Sprague-Dawley rats: effects of anxiolytic and anxiogenic agents. Pharmacol. Biochem. Behav. 97, 406–415. 10.1016/j.pbb.2010.09.013 20869983 PMC3006066

[B20] Calhoon G. G. Tye K. M. (2015). Resolving the neural circuits of anxiety. Nat. Neurosci. 18, 1394–1404. 10.1038/nn.4101 26404714 PMC7575249

[B21] Canton-Josh J. E. Qin J. Salvo J. Kozorovitskiy Y. (2022). Dopaminergic regulation of vestibulo-cerebellar circuits through unipolar brush cells. eLife 11:e76912. 10.7554/eLife.76912 35476632 PMC9106328

[B22] Carta I. Chen C. H. Schott A. L. Dorizan S. Khodakhah K. (2019). Cerebellar modulation of the reward circuitry and social behavior. Science 11:eaav0581. 10.1126/science.aav0581 30655412 PMC6711161

[B23] Cartford M. C. Samec A. Fister M. Bickford P. C. (2004). Cerebellar norepinephrine modulates learning of delay classical eyeblink conditioning: evidence for post-synaptic signaling via PKA. Learn. Mem. 11, 732–737. 10.1101/lm.83104 15537737 PMC534701

[B24] Cassano G. B. Baldini Rossi N. Pini S. (2002). Psychopharmacology of anxiety disorders. Dialogues Clin. Neurosci. 4, 271–285. 10.31887/DCNS.2002.4.3/gcassano 22033867 PMC3181684

[B25] Cassimjee N. Fouche J. P. Burnett M. Lochner C. Warwick J. Dupont P.. (2010). Changes in regional brain volumes in social anxiety disorder following 12 weeks of treatment with escitalopram. Metab. Brain Dis. 25, 369–374. 10.1007/s11011-010-9218-6 21063760

[B26] Cavdar S. Ozgur M. Kuvvet Y. Bay H. H. (2018). The cerebello-hypothalamic and hypothalamo-cerebellar pathways via superior and middle cerebellar peduncle in the rat. Cerebellum 17, 517–524. 10.1007/s12311-018-0938-1 29637507

[B27] Chambers W. W. Sprague J. M. (1955). Functional localization in the cerebellum. II. somatotopic organization in cortex and nuclei. Arch. Neurol. Psychiatry 74, 653–680. 10.1001/archneurpsyc.1955.02330180071008 13268132

[B28] Chao O. Y. Zhang H. Pathak S. S. Huston J. P. Yang Y. M. (2021). Functional convergence of motor and social processes in lobule IV/V of the mouse cerebellum. Cerebellum 20, 836–852. 10.1007/s12311-021-01246-7 33661502 PMC8417139

[B29] Chen X. Du Y. Broussard G. J. Kislin M. Yuede C. M. Zhang S.. (2022). Transcriptomic mapping uncovers Purkinje neuron plasticity driving learning. Nature 605, 722–727. 10.1038/s41586-022-04711-3 35545673 PMC9887520

[B30] Costa J. N. Serafim K. R. Gianlorenco A. C. Mattioli R. (2013). Low-dose thioperamide injected into the cerebellar vermis of mice immediately after exposure to the elevated plus-maze impairs their avoidance behavior on re-exposure to the apparatus. Braz. J. Med. Biol. Res. 46, 943–948. 10.1590/1414-431X20133179 24270913 PMC3854336

[B31] Cutando L. Puighermanal E. Castell L. Tarot P. Belle M. Bertaso F.. (2022). Cerebellar dopamine D2 receptors regulate social behaviors. Nat. Neurosci. 25, 900–911. 10.1038/s41593-022-01092-8 35710984

[B32] D’Angelo E. Casali S. (2012). Seeking a unified framework for cerebellar function and dysfunction: from circuit operations to cognition. Front. Neural Circuits 6:116. 10.3389/fncir.2012.00116 23335884 PMC3541516

[B33] Depping M. S. Schmitgen M. M. Kubera K. M. Wolf R. C. (2018). Cerebellar contributions to major depression. Front. Psychiatry 6:634. 10.3389/fpsyt.2018.00634 30555360 PMC6281716

[B34] Dietrichs E. (1984). Cerebellar autonomic function: direct hypothalamocerebellar pathway. Science 223, 591–593. 10.1126/science.6198719 6198719

[B35] Dietrichs E. Haines D. E. (1986). Do the same hypothalamic neurons project to both amygdala and cerebellum? Brain Res. 364, 241–248. 10.1016/0006-8993(86)90836-x 2418916

[B36] Dieudonne S. (1998). Submillisecond kinetics and low efficacy of parallel fibre-Golgi cell synaptic currents in the rat cerebellum. J. Physiol. 510, 845–866. 10.1111/j.1469-7793.1998.845bj.x 9660898 PMC2231065

[B37] Doruyter A. Lochner C. Jordaan G. P. Stein D. J. Dupont P. Warwick J. M. (2016). Resting functional connectivity in social anxiety disorder and the effect of pharmacotherapy. Psychiatry Res. Neuroimaging 251, 34–44. 10.1016/j.pscychresns.2016.04.009 27111811

[B38] Dugué G. P. Dumoulin A. Triller A. Dieudonné S. (2005). Target-dependent use of coreleased inhibitory transmitters at central synapses. J. Neurosci. 25, 6490–6498. 10.1523/JNEUROSCI.1500-05.2005 16014710 PMC6725433

[B39] Engel K. Bandelow B. Gruber O. Wedekind D. (2009). Neuroimaging in anxiety disorders. J. Neural Transm. (Vienna) 116, 703–716. 10.1007/s00702-008-0077-9 18568288 PMC2694920

[B40] Etkin A. Prater K. E. Schatzberg A. F. Menon V. Greicius M. D. (2009). Disrupted amygdalar subregion functional connectivity and evidence of a compensatory network in generalized anxiety disorder. Arch. Gen. Psychiatry 66, 1361–1372. 10.1001/archgenpsychiatry.2009.104 19996041 PMC12553334

[B41] Evans K. C. Wright C. I. Wedig M. M. Gold A. L. Pollack M. H. Rauch S. L. (2008). A functional MRI study of amygdala responses to angry schematic faces in social anxiety disorder. Depress. Anxiety 25, 496–505. 10.1002/da.20347 17595018

[B42] Fernandes C. E. M. Serafim K. R. Gianlorenco A. C. L. Mattioli R. (2017). Intra-vermis H4 receptor agonist impairs performance in anxiety- and fear-mediated models. Brain Res. Bull. 135, 179–184. 10.1016/j.brainresbull.2017.10.014 29097243

[B43] Frontera J. L. Baba Aissa H. Sala R. W. Mailhes-Hamon C. Georgescu I. A. Léna C.. (2020). Bidirectional control of fear memories by cerebellar neurons projecting to the ventrolateral periaqueductal grey. Nat. Commun. 11:5207. 10.1038/s41467-020-18953-0 33060630 PMC7566591

[B44] Fujita H. Kodama T. du Lac S. (2020). Modular output circuits of the fastigial nucleus for diverse motor and nonmotor functions of the cerebellar vermis. eLife 9:e58613. 10.7554/eLife.58613 32639229 PMC7438114

[B45] Furmark T. Tillfors M. Marteinsdottir I. Fischer H. Pissiota A. Långström B.. (2002). Common changes in cerebral blood flow in patients with social phobia treated with citalopram or cognitive-behavioral therapy. Arch. Gen. Psychiatry 59, 425–433. 10.1186/s40337-022-00730-7 11982446

[B46] Gianlorenco A. C. Canto-de-Souza A. Mattioli R. (2011). Microinjection of histamine into the cerebellar vermis impairs emotional memory consolidation in mice. Brain Res. Bull. 86, 134–138. 10.1016/j.brainresbull.2011.05.014 21664441

[B47] Gianlorenco A. C. Serafim K. R. Canto-de-Souza A. Mattioli R. (2012). Emotional memory consolidation impairment induced by histamine is mediated by H1 but not H2 receptors. Brain Res. Bull. 89, 197–202. 10.1016/j.brainresbull.2012.09.003 22986235

[B48] Godlevsky L. S. Muratova T. N. Kresyun N. V. van Luijtelaar G. Coenen A. M. (2014). Anxiolytic and antidepressive effects of electric stimulation of the paleocerebellar cortex in pentylenetetrazol kindled rats. Acta Neurobiol. Exp. (Wars) 74, 456–464. 25576976 10.55782/ane-2014-2008

[B49] Gould T. J. (1998). β-Adrenergic involvement in acquisition vs. extinction of a classically conditioned eye blink response in rabbits. Brain Res. 780, 174–177. 9497096

[B50] Ha S. Lee D. Cho Y. S. Chung C. Yoo Y. E. Kim J.. (2016). Cerebellar shank2 regulates excitatory synapse density, motor coordination and specific repetitive and anxiety-like behaviors. J. Neurosci. 36, 12129–12143. 10.1523/JNEUROSCI.1849-16.2016 27903723 PMC6601982

[B51] Hilber P. Lorivel T. Delarue C. Caston J. (2004). Stress and anxious-related behaviors in Lurcher mutant mice. Brain Res. 1003, 108–112. 10.1016/j.brainres.2004.01.008 15019569

[B52] Hioki H. Fujiyama F. Taki K. Tomioka R. Furuta T. Tamamaki N.. (2003). Differential distribution of vesicular glutamate transporters in the rat cerebellar cortex. Neuroscience 117, 1–6. 10.1016/s0306-4522(02)00943-0 12605886

[B53] Ito M. (2002). Historical review of the significance of the cerebellum and the role of Purkinje cells in motor learning. Ann. N Y Acad. Sci. 978, 273–288. 10.1111/j.1749-6632.2002.tb07574.x 12582060

[B54] Ito M. (2006). Cerebellar circuitry as a neuronal machine. Prog. Neurobiol. 78, 272–303. 10.1016/j.pneurobio.2006.02.006 16759785

[B55] Ito M. Nisimaru N. (2014). Cerebellar control of defense reactions under orexin-mediated neuromodulation as a model of cerebellohypothalamic interaction. AIMS Neurosci. 1, 89–95. 10.3934/Neuroscience.2014.1.89

[B57] Kashyap R. Eng G. K. Bhattacharjee S. Gupta B. Ho R. Ho C. S. H.. (2021). Individual-fMRI-approaches reveal cerebellum and visual communities to be functionally connected in obsessive compulsive disorder. Sci. Rep. 11:1354. 10.1038/s41598-020-80346-6 33446780 PMC7809273

[B58] Ke J. Zhang L. Qi R. Li W. Hou C. Zhong Y.. (2016). A longitudinal fMRI investigation in acute post-traumatic stress disorder (PTSD). Acta Radiol. 57, 1387–1395. 10.1177/0284185115585848 25995310

[B59] Kelp A. Koeppen A. H. Petrasch-Parwez E. Calaminus C. Bauer C. Portal E.. (2013). A novel transgenic rat model for spinocerebellar ataxia type 17 recapitulates neuropathological changes and supplies in vivo imaging biomarkers. J. Neurosci. 33, 9068–9081. 10.1523/JNEUROSCI.5622-12.2013 23699518 PMC6705027

[B60] Kim J. Augustine G. J. (2021). Molecular layer interneurons: key elements of cerebellar network computation and behavior. Neuroscience 462, 22–35. 10.1016/j.neuroscience.2020.10.008 33075461

[B62] Kim J. E. Chae S. Kim S. Jung Y. J. Kang M. G. Heo W.. (2021). Cerebellar 5HT-2A receptor mediates stress-induced onset of dystonia. Sci. Adv. 7:eabb5735. 10.1126/sciadv.abb5735 33658190 PMC7929497

[B61] Kim J. Lee S. Tsuda S. Zhang X. Asrican B. Gloss B.. (2014). Optogenetic mapping of cerebellar inhibitory circuitry reveals spatially biased coordination of interneurons via electrical synapses. Cell Rep. 7, 1601–1613. 10.1016/j.celrep.2014.04.047 24857665 PMC4107211

[B63] Klein A. P. Ulmer J. L. Quinet S. A. Mathews V. Mark L. P. (2016). Nonmotor functions of the cerebellum: an introduction. Am. J. Neuroradiol. 37, 1005–1009. 10.3174/ajnr.A4720 26939633 PMC7963530

[B64] Koeppen A. H. (2018). The neuropathology of the adult cerebellum. Handb. Clin. Neurol. 154, 129–149. 10.1016/B978-0-444-63956-1.00008-4 29903436 PMC6279249

[B65] Kondo M. Sears T. A. Sadakane K. Nisimaru N. (1998). Vagal afferent projections to lobule VIIa of the rabbit cerebellar vermis related to cardiovascular control. Neurosci. Res. 30, 111–117. 10.1016/s0168-0102(97)00112-0 9579644

[B66] Koutsikou S. Crook J. J. Earl E. V. Leith J. L. Watson T. C. Lumb B. M.. (2014). Neural substrates underlying fear-evoked freezing: the periaqueductal grey-cerebellar link. J. Physiol. 592, 2197–2213. 10.1113/jphysiol.2013.268714 24639484 PMC4027863

[B67] LeDoux J. E. Pine D. S. (2016). Using neuroscience to help understand fear and anxiety: a two-system framework. Am. J. Psychiatry 173, 1083–1093. 10.1176/appi.ajp.2016.16030353 27609244

[B68] Liao W. Qiu C. Gentili C. Walter M. Pan Z. Ding J.. (2010). Altered effective connectivity network of the amygdala in social anxiety disorder: a resting-state FMRI study. PLoS One 5:e15238. 10.1371/journal.pone.0015238 21203551 PMC3008679

[B69] Longley M. Ballard J. Andres-Alonso M. Varatharajah R. C. Cuthbert H. Yeo C. H. (2021). A patterned architecture of monoaminergic afferents in the cerebellar cortex: noradrenergic and serotonergic fibre distributions within lobules and parasagittal zones. Neuroscience 462, 106–121. 10.1016/j.neuroscience.2020.09.001 32949672

[B70] Lorivel T. Gras M. Hilber P. (2010). Effects of corticosterone synthesis inhibitor metyrapone on anxiety-related behaviors in Lurcher mutant mice. Physiol. Behav. 101, 309–314. 10.1016/j.physbeh.2010.05.011 20684068

[B71] Lupo M. Siciliano L. Leggio M. (2019). From cerebellar alterations to mood disorders: a systematic review. Neurosci. Biobehav. Rev. 103, 21–28. 10.1016/j.neubiorev.2019.06.008 31195001

[B72] Martin E. I. Ressler K. J. Binder E. Nemeroff C. B. (2009). The neurobiology of anxiety disorders: brain imaging, genetics and psychoneuroendocrinology. Psychiatr. Clin. North Am. 32, 549–575. 10.1016/j.psc.2009.05.004 19716990 PMC3684250

[B73] McCall J. G. Siuda E. R. Bhatti D. L. Lawson L. A. McElligott Z. A. Stuber G. D.. (2017). Locus coeruleus to basolateral amygdala noradrenergic projections promote anxiety-like behavior. eLife 6:e18247. 10.7554/eLife.18247 28708061 PMC5550275

[B74] Monnier C. Lalonde R. (1995). Elevated (+)-maze and hole-board exploration in lurcher mutant mice. Brain Res. 702, 169–172. 10.1016/0006-8993(95)01036-5 8846072

[B75] Morel C. Montgomery S. E. Li L. Durand-de Cuttoli R. Teichman E. M. Juarez B.. (2022). Midbrain projection to the basolateral amygdala encodes anxiety-like but not depression-like behaviors. Nat. Commun. 13:1532. 10.1038/s41467-022-29155-1 35318315 PMC8940900

[B76] Moreno-Rius J. (2018). The cerebellum in fear and anxiety-related disorders. Prog. Neuropsychopharmacol. Biol. Psychiatry 85, 23–32. 10.1016/j.pnpbp.2018.04.002 29627508

[B77] Morton S. M. Bastian A. J. (2004). Cerebellar control of balance and locomotion. Neuroscientist 10, 247–259. 10.1177/1073858404263517 15155063

[B78] Moulédous L. Roullet P. Guiard B. P. (2018). “Brain circuits regulated by the 5-HT2A Receptor: Behavioural Consequences on Anxiety and Fear Memory,” in 5-HT2A Receptors in the Central Nervous System, eds. Di Giovanni B. P. Fay G. (Cham: Springer International Publishing), 231–258.

[B79] Nilaweera W. U. Zenitsky G. D. Bracha V. (2006). Inactivation of cerebellar output axons impairs acquisition of conditioned eyeblinks. Brain Res. 1122, 143–153. 10.1016/j.brainres.2006.08.127 17067561 PMC1850997

[B80] Novello M. Bosman L. W.J. De Zeeuw C. I. (2022). A systematic review of direct outputs from the cerebellum to the brainstem and diencephalon in mammals. Cerebellum 10.1007/s12311-022-01499-w. [Online ahead of print]. 36575348 PMC10864519

[B81] Phillips J. R. Hewedi D. H. Eissa A. M. Moustafa A. A. (2015). The cerebellum and psychiatric disorders. Front. Public Health 3:66. 10.3389/fpubh.2015.00066 26000269 PMC4419550

[B82] Pierce E. Hoddevik G. Walberg F. (1977). The cerebellar projection from the raphe nuclei in the cat as studied with the method of retrograde transport of horseradish peroxidase. Anat. Embryol. (Berl) 152, 73–87. 10.1007/BF00341436 605998

[B83] Popa L. S. Streng M. L. Ebner T. J. (2019). Purkinje cell representations of behavior: diary of a busy neuron. Neuroscientist 25, 241–257. 10.1177/1073858418785628 29985093 PMC6509027

[B84] Prasko J. Horacek J. Zalesky R. Kopecek M. Novak T. Paskova B.. (2004). The change of regional brain metabolism (18FDG PET) in panic disorder during the treatment with cognitive behavioral therapy or antidepressants. Neuro. Endocrinol. Lett. 25, 340–348. Available online at: https://www.nel.edu/the-change-of-regional-brain-metabolism-18fdg-pet-in-panic-disorder-during-the-treatment-with-cognitive-behavioral-therapy-or-antidepressants-2045/. 15580167

[B85] Reeber S. L. Otis T. S. Sillitoe R. V. (2013). New roles for the cerebellum in health and disease. Front. Syst. Neurosci. 7:83. 10.3389/fnsys.2013.00083 24294192 PMC3827539

[B86] Richter S. Schoch B. Kaiser O. Groetschel H. Dimitrova A. Hein-Kropp C.. (2005). Behavioral and affective changes in children and adolescents with chronic cerebellar lesions. Neurosci. Lett. 381, 102–107. 10.1016/j.neulet.2005.02.011 15882798

[B87] Rieubland S. Roth A. Hausser M. (2014). Structured connectivity in cerebellar inhibitory networks. Neuron 81, 913–929. 10.1016/j.neuron.2013.12.029 24559679 PMC3988957

[B88] Riklan M. Cullinan T. Cooper I. S. (1977). Tension reduction and alerting in man following chronic cerebellar stimulation for the relief of spasticity or intractable seizures. J. Nerv. Ment. Dis. 164, 176–181. 10.1097/00005053-197703000-00003 300098

[B89] Roy A. K. Fudge J. L. Kelly C. Perry J. S. Daniele T. Carlisi C.. (2013). Intrinsic functional connectivity of amygdala-based networks in adolescent generalized anxiety disorder. J. Am. Acad. Child Adolesc. Psychiatry 52, 290–299.e2. 10.1016/j.jaac.2012.12.010 23452685 PMC3760686

[B90] Rudolph S. Guo C. Pashkovski S. L. Osorno T. Gillis W. F. Krauss J. M.. (2020). Cerebellum-Specific deletion of the GABA_A_ receptor δ subunit leads to sex-specific disruption of behavior. Cell Rep. 33:108338. 10.1016/j.celrep.2020.108338 33147470 PMC7700496

[B91] Sacchetti B. Baldi E. Lorenzini C. A. Bucherelli C. (2002). Cerebellar role in fear-conditioning consolidation. Proc. Natl. Acad. Sci. U S A 99, 8406–8411. 10.1073/pnas.112660399 12034877 PMC123080

[B92] Sacchetti B. Sacco T. Strata P. (2007). Reversible inactivation of amygdala and cerebellum but not perirhinal cortex impairs reactivated fear memories. Eur. J. Neurosci. 25, 2875–2884. 10.1111/j.1460-9568.2007.05508.x 17466022

[B93] Sakai Y. Kumano H. Nishikawa M. Sakano Y. Kaiya H. Imabayashi E.. (2005). Cerebral glucose metabolism associated with a fear network in panic disorder. Neuroreport 16, 927–931. 10.1097/00001756-200506210-00010 15931063

[B94] Schmahmann J. D. Caplan D. (2006). Cognition, emotion and the cerebellum. Brain 129, 290–292. 10.1093/brain/awh729 16434422

[B95] Schmahmann J. D. Weilburg J. B. Sherman J. C. (2007). The neuropsychiatry of the cerebellum - insights from the clinic. Cerebellum 6, 254–267. 10.1080/14734220701490995 17786822

[B96] Schutter D. J. (2012). The cerebello-hypothalamic-pituitary-adrenal axis dysregulation hypothesis in depressive disorder. Med. Hypotheses 79, 779–783. 10.1016/j.mehy.2012.08.027 22999737

[B97] Schutter D. J. L. G. van Honk J. (2009). The cerebellum in emotion regulation: a repetitive transcranial magnetic stimulation study. Cerebellum 8, 28–34. 10.1007/s12311-008-0056-6 18855096

[B98] Schutter D. J. L. G. van Honk J. d’Alfonso A. A. L. Peper J. S. Panksepp J. (2003). High frequency repetitive transcranial magnetic over the medial cerebellum induces a shift in the prefrontal electroencephalography gamma spectrum: a pilot study in humans. Neurosci. Lett. 336, 73–76. 10.1016/s0304-3940(02)01077-7 12499043

[B99] Snider R. S. Maiti A. (1976). Cerebellar contributions to the Papez circuit. J. Neurosci. Res. 2, 133–146. 10.1002/jnr.490020204 950678

[B100] Stoodley C. J. Schmahmann J. D. (2010). Evidence for topographic organization in the cerebellum of motor control versus cognitive and affective processing. Cortex 46, 831–844. 10.1016/j.cortex.2009.11.008 20152963 PMC2873095

[B101] Stoodley C. J. Schmahmann J. D. (2018). “Chapter 4 - Functional topography of the human cerebellum,” in Handbook of Clinical Neurology, eds Manto M. Huisman T. A. G. M. (Amsterdam, Netherlands: Elsevier), 59–70. 10.1016/B978-0-444-63956-1.00004-7 29903452

[B102] Strick P. L. Dum R. P. Fiez J. A. (2009). Cerebellum and nonmotor function. Annu. Rev. Neurosci. 32, 413–434. 10.1146/annurev.neuro.31.060407.125606 19555291

[B103] Supple W. F. Jr. (1993). Hypothalamic modulation of Purkinje cell activity in the anterior cerebellar vermis. Neuroreport 4, 979–982. 10.1097/00001756-199307000-00036 8369494

[B104] Supple W. F. Jr. Leaton R. N. Fanselow M. S. (1987). Effects of cerebellar vermal lesions on species-specific fear responses, neophobia and taste-aversion learning in rats. Physiol. Behav. 39, 579–586. 10.1016/0031-9384(87)90156-9 3588702

[B105] Szucs D. Ioannidis J. P.A. (2020). Sample size evolution in neuroimaging research: an evaluation of highly-cited studies (1990–2012) and of latest practices (2017–2018) in high-impact journals. Neuroimage 221:117164. 10.1016/j.neuroimage.2020.117164 32679253

[B106] Talati A. Pantazatos S. P. Hirsch J. Schneier F. (2015). A pilot study of gray matter volume changes associated with paroxetine treatment and response in social anxiety disorder. Psychiatry Res. 231, 279–285. 10.1016/j.pscychresns.2015.01.008 25659476 PMC4363180

[B107] Talati A. Pantazatos S. P. Schneier F. R. Weissman M. M. Hirsch J. (2013). Gray matter abnormalities in social anxiety disorder: primary, replication and specificity studies. Biol. Psychiatry 73, 75–84. 10.1016/j.biopsych.2012.05.022 22748614 PMC3465490

[B108] Thach W. T. Goodkin H. P. Keating J. G. (1992). The cerebellum and the adaptive coordination of movement. Annu. Rev. Neurosci. 15, 403–442. 10.1146/annurev.ne.15.030192.002155 1575449

[B109] Timmann D. Drepper J. Frings M. Maschke M. Richter S. Gerwig M.. (2010). The human cerebellum contributes to motor, emotional and cognitive associative learning. A review. Cortex 46, 845–857. 10.1016/j.cortex.2009.06.009 19665115

[B110] Tovote P. Fadok J. P. Luthi A. (2015). Neuronal circuits for fear and anxiety. Nat. Rev. Neurosci. 16, 317–331. 10.1038/nrn3945 25991441

[B111] Tucker L. B. McCabe J. T. (2017). Behavior of male and female C57BL/6J mice is more consistent with repeated trials in the elevated zero maze than in the elevated plus maze. Front. Behav. Neurosci. 11:13. 10.3389/fnbeh.2017.00013 28184191 PMC5266707

[B112] Uemura T. Lee S.-J. Yasumura M. Takeuchi T. Yoshida T. Ra M.. (2010). Trans-synaptic interaction of gluRδ2 and neurexin through cbln1 mediates synapse formation in the cerebellum. Cell 141, 1068–1079. 10.1016/j.cell.2010.04.035 20537373

[B113] Vogel M. W. Caston J. Yuzaki M. Mariani J. (2007). The Lurcher mouse: fresh insights from an old mutant. Brain Res. 1140, 4–18. 10.1016/j.brainres.2005.11.086 16412991

[B114] Wang S. S. Kloth A. D. Badura A. (2014). The cerebellum, sensitive periods and autism. Neuron 83, 518–532. 10.1016/j.neuron.2014.07.016 25102558 PMC4135479

[B115] Warwick J. M. Carey P. Jordaan G. P. Dupont P. Stein D. J. (2008). Resting brain perfusion in social anxiety disorder: a voxel-wise whole brain comparison with healthy control subjects. Prog. Neuropsychopharmacol. Biol. Psychiatry 32, 1251–1256. 10.1016/j.pnpbp.2008.03.017 18485554

[B116] Xu T. Zhao Q. Wang P. Fan Q. Chen J. Zhang H.. (2019). Altered resting-state cerebellar-cerebral functional connectivity in obsessive-compulsive disorder. Psychol. Med. 49, 1156–1165. 10.1017/S0033291718001915 30058519

[B117] Zangrossi H. Del Ben C. M. Graeff F. G. Guimarães F. S. (2020). “"Serotonin in panic and anxiety disorders,” in Handbook of Behavioral Neuroscience, eds Müller C. P. Cunningham K. A. (London, United Kingdom: Elsevier), 611–633.

[B118] Zhang H. Wang B. Li K. Wang X. Li X. Zhu J.. (2019). Altered functional connectivity between the cerebellum and the cortico-striato-thalamo-cortical circuit in obsessive-compulsive disorder. Front. Psychiatry 10:522. 10.3389/fpsyt.2019.00522 31396115 PMC6667674

[B119] Zhang Q. Wu J. F. Shi Q. L. Li M. Y. Wang C. J. Wang X.. (2019). The neuronal activation of deep cerebellar nuclei is essential for environmental enrichment-induced post-stroke motor recovery. Aging Dis. 10, 530–543. 10.14336/AD.2018.1220 31164998 PMC6538218

[B121] Zhu L. Scelfo B. Hartell N. A. Strata P. Sacchetti B. (2007). The effects of fear conditioning on cerebellar LTP and LTD. Eur. J. Neurosci. 26, 219–227. 10.1111/j.1460-9568.2007.05632.x 17573921

[B120] Zhu J. N. Yung W. H. Kwok-Chong Chow B. Chan Y. S. Wang J. J. (2006). The cerebellar-hypothalamic circuits: potential pathways underlying cerebellar involvement in somatic-visceral integration. Brain Res. Rev. 52, 93–106. 10.1016/j.brainresrev.2006.01.003 16497381

